# DNA of *Theileria orientalis*, *T. equi* and *T. capreoli* in stable flies (*Stomoxys calcitrans*)

**DOI:** 10.1186/s13071-020-04041-1

**Published:** 2020-04-09

**Authors:** Sándor Hornok, Nóra Takács, Sándor Szekeres, Krisztina Szőke, Jenő Kontschán, Gábor Horváth, László Sugár

**Affiliations:** 1 Department of Parasitology and Zoology, University of Veterinary Medicine, Budapest, Hungary; 2 Plant Protection Institute, Centre for Agricultural Researches, Budapest, Hungary; 3 Veterinary Authority, Csurgó, Hungary; 4 Department of Game Management and Ethology, Faculty of Agricultural and Environmental Sciences, University of Kaposvár, Kaposvár, Hungary

**Keywords:** Muscidae, Blood-sucking, Mechanical vector, Male, Female, Gut, Diverticulum, *Haematobia stimulans*

## Abstract

**Background:**

From a veterinary-medical point of view, the stable fly, *Stomoxys calcitrans*, is perhaps the economically most important blood-sucking muscoid fly species (Diptera: Muscidae), owing to its worldwide occurrence, frequently high local abundance, direct harm caused to livestock, pet animals and humans, as well as its vector role. Considering the latter in the context of protozoan parasites, the stable fly is a mechanical vector of trypanosomes and *Besnoitia besnoiti*. However, its role as a vector of piroplasms appears to be seldom studied, despite old data suggesting mechanical transmission of babesiae by dipteran flies.

**Methods:**

In this study 395 stable flies (and one *Haematobia stimulans*) were collected at a cattle farm with known history of bovine theileriosis, and at further nine, randomly chosen locations in Hungary. These flies were separated according to sex (30 of them also cut into two parts: the head with mouthparts and the thorax-abdomen), followed by individual DNA extraction, then screening for piroplasms by PCR and sequencing.

**Results:**

In stable flies, *Theileria orientalis* and *T. capreoli* were identified at the cattle farm and *T. equi* was identified in three other locations. At the cattle farm, significantly more male stable flies carried piroplasm DNA than females. There was no significant difference between the ratio of PCR-positive flies between the stable (void of cattle for at least two hours) and the pen on the pasture with cattle at the time of sampling. Among dissected flies (29 *S. calcitrans* and 1 *H. stimulans*), exclusively the thoracic-abdominal parts were PCR-positive, whereas the head and mouthparts remained negative.

**Conclusions:**

*Theileria* DNA is detectable in stable flies, in the case of *T. orientalis* at least for two hours after blood-feeding, and in the case of *T. capreoli* also in the absence of infected hosts (i.e. roe deer). Male flies rather than females, and thoracic-abdominal (most likely crop) contents rather than mouthparts may pose a risk of mechanical transmission. These data suggest that it is worth to study further the vector role of stable flies in the epidemiology of theilerioses, in which not the immediate, but rather the delayed type transmission seems possible.
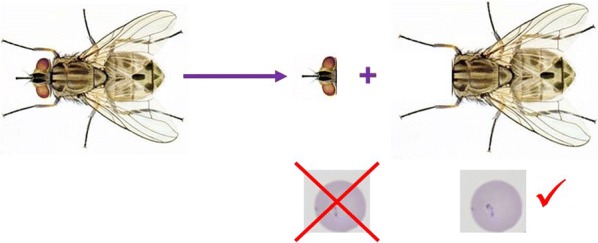

## Introduction

The stable fly, *Stomoxys calcitrans*, is a muscoid fly species (Diptera: Muscidae) with a worldwide distribution and increasingly recognized veterinary and medical significance [1]. Although stable flies attack a wide range of domestic and wild animals (including rats, guinea pigs, rabbits, monkeys, dogs, horses, camels, goats and pelicans), as well as humans, cattle are their main hosts [2]. Both sexes feed on blood, causing annoyance, blood loss and wounds [3]. Furthermore, *Stomoxys* flies are mechanical vectors of pathogens present in the blood and skin of their hosts, because in case of interrupted feeding, they can re-start their blood meal on another host [1]. When injecting saliva prior to blood-sucking, the stable fly can inoculate some infected blood remaining in its mouthparts [1]. Besides this immediate transmission, it was observed that stable flies may keep some blood in their crop (oesophageal diverticulum), which offers a friendly environment for pathogens that could be regurgitated during the next blood meal; thus a delayed transmission is also possible [1].

Among the pathogens known to be transmitted by stable flies, there are numerous economically significant viruses, rickettsiae and other bacteria. Considering protozoan parasites, the stable fly is a mechanical vector of trypanosomes and *Besnoitia besnoiti* [1]. Importantly, transmission experiments involving *Trypanosoma evansi* demonstrated a peculiar capacity of the stable fly for delayed transmission, in contrast to tabanids [1].

Piroplasms (Apicomplexa: Piroplasmida) are haemotropic protozoan parasites [4]. Among them, *Babesia* and *Theileria* species have soft and hard ticks (Acari: Ixodidae, Argasidae) as biological vectors, but old data also attest that blood-sucking flies, including members of the family Tabanidae and of the genus *Stomoxys*, can act as mechanical vectors in their transmission [4]. In particular, it has been demonstrated that members of the *Theileria orientalis* complex are capable of being mechanically transferred between cattle by intravenous inoculation with small volumes of blood [5] as well as by biting arthropods (by *Tabanus trigeminus* [6]; and by the sucking louse *Linognathus vituli* [7]). Despite of this, the role of stable flies in transmitting piroplasms appears to be seldom investigated.

In Hungary, the occurrence of *T. orientalis* has recently been demonstrated, but attempts to find the corresponding tick vector(s) failed so far [8]. This was the main reason for initiating the present study. In addition, when *Babesia* DNA was detected in the feces of bats, this was partly explained by the predominance of stable flies in the diet of some bat species [9], but the actual possibility of piroplasm carriage by these flies remains to be elucidated. Thus, the aims of the present study were to investigate: (i) if DNA of *Babesia* or *Theileria* is present in stable flies; (ii) if parasite DNA can also be detected in flies a few hours after their access to cattle or in the absence of nearby hosts (which could have provided infectious source); and (iii) to ascertain whether mouthpart-associated immediate or crop-associated delayed transmission is more likely.

## Methods

### Sample collection

Altogether 239 blood-sucking muscoid flies were collected at a cattle farm (Pásztó, 47°55’34.5”N, 19°40’49.8”E in northern Hungary) with known occurrence of *Theileria orientalis*, near the end of each month between August and October, 2017. At this location, flies were collected from 9:00 to 10:30 h at the main stable (from which all cattle were transferred to pens on the pasture before 7:00 h), and also in the pens near the cattle from 10:30 to 12:00 h. The pen and pasture are at a distance of *c.* 0.5 km from the stable.

**Fig. 1:**
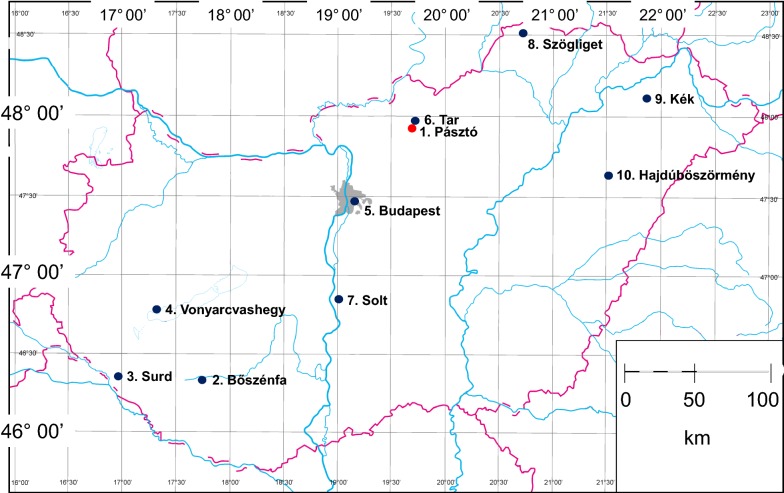
Location, serial number and name of sampling sites. The red dot indicates the place of the cattle farm with repeated sampling

In addition, 157 blood-sucking muscoid flies were collected in further nine, randomly chosen locations (Fig. 1) with or without the presence of domestic or game animals (Table 1), between October 2017 and August 2019. Flies were caught with commercial fishing nets, and if intact, preserved in 70% ethanol. Species identification (395 *Stomoxys calcitrans* and 1 *Haematobia stimulans*) and sexing was based on standard keys [10].

**Table 1:** Results of molecular analyses for piroplasm DNA in *Stomoxys calcitrans* according to collection site, month and sex

Locality	Habitat characteristics	Month	PCR-positive among all tested		Result of sequencing		GenBank ID
			Males	Females	Species (*n*)	Identity (%)	
1	Stable (void of cattle for > 2 h)	August	0/2	1/14	*T. orientalis* (1×)	100	MN611176
		September	20/55	2/4	*T. orientalis* (22×)	100	MN611176
		October	14/30	15/45	*T. orientalis* (29×)	100	MN611176
	Pen on pasture (with cattle)	August	1/2	2/14	*T. capreoli*^a^ (1×)	100	MN611178
					*T. orientalis* (2×)	100	MN611176
		September	11/22	2/10	*T. orientalis* (13×)	100	MN611176
		October	14/31	5/9	*T. orientalis* (19×)	100	MN611176
2	Farm with horses, game	October	0/2	2/21	*T. equi* (2×)	100	MN611177
3	Farm with cattle	October	0/3	0/5	–	–	–
4	Lake shore (beach)	August	0/1	0/3	–	–	–
5	Garden (in capital city)	November	0/1	–	–	–	–
6	Garden (in a village)	September	0/1	–	–	–	–
7	Livestock reserve with horses and ruminants	June	1/25	0/59	*T. equi* (1×)	100	MN611177
8	Garden with a horse	July	1/4	1/5	*T. equi* (2×)	100	MN611177
9	Garden (in a village)	September	0/2	–	–	–	–
10	Garden (in a village)	September	0/17	0/8	–	–	–

^a^Isolate “capreoli-CE1” (KY308178)

Flies were separated according to sex, disinfected on their surface with sequential washing for 15 s in three Petri dishes (containing 10% sodium-hypochlorite, tap water or distilled water, respectively), then air-dried. Thirty flies were dissected into two parts (the head with the mouthparts and the thorax-abdomen) with sterile scalpel blades. These dissected flies included both males (*n* = 20) and females (*n* = 10), collected in the pen on pasture, in the middle of the main sampling period (September 2017) at the main site (No. 1 in Fig. 1).

### DNA extraction, PCR and sequencing

DNA was extracted individually from each fly or their two parts, by using the QIAamp DNA Mini Kit (Qiagen, Hilden, Germany) according to the manufacturerʼs instructions, including an overnight digestion in tissue lysis buffer and 6.6% Proteinase K at 56 °C. An extraction control (100 μl phosphate-buffered saline processed together with the samples) was also added to each set of 23 samples, for monitoring cross-contamination.

The conventional PCR used for the detection of piroplasms was modified from Casati et al. [11], as reported in Hornok et al. [12]. This method amplifies an approximately 500 bp long fragment of the *18S* rRNA gene of *Babesia* and *Theileria* species with the primers BJ1 (forward: 5’-GTC TTG TAA TTG GAA TGA TGG-3’) and BN2 (reverse: 5’-TAG TTT ATG GTT AGG ACT ACG-3’). Purification and sequencing of the PCR products were performed by Biomi Ltd. (Gödöllő, Hungary). Obtained sequences were manually edited, then aligned and compared to reference GenBank sequences by the BLASTn program (https://blast.ncbi.nlm.nih.gov). Representative sequences were submitted to the GenBank database under the accession numbers MN611176- MN611178.

### Statistical analysis

Prevalence rates were compared with Fisherʼs exact test, and differences were considered significant if *P* < 0.05.

## Results

### Piroplasms in stable flies at the cattle farm (locality 1)

From August to October 36.6% of stable flies (87/238) were PCR-positive for piroplasms (Table 1). Males carried piroplasm DNA significantly more frequently than females (60 of 142 *vs* 27 of 96, respectively; *P* = 0.028). Compared to August, the ratio of PCR-positive flies was significantly higher both in September (4 of 32 *vs* 35 of 91, respectively; *P* = 0.008) and in October (4 of 32 *vs* 48 of 115, respectively; *P* = 0.002). However, there was no significant difference in the ratio of PCR-positive flies between the stable (void of cattle) and the pen on the pasture with cattle at the time of sampling (52 of 150 *vs* 35 of 88; *P* = 0.48; Table 1). In addition, the single specimen of *Haematobia stimulans* was also PCR-positive for piroplasms.

Considering dissected flies (29 *S. calcitrans* and 1 *H. stimulans*), in all cases exclusively the body (thoracic-abdominal part) of flies was PCR-positive, whereas the head and mouthparts remained negative. This was a highly significant association (*P* < 0.0001).

Sequencing identified *T. orientalis* in the majority (86 of 87) of the PCR-positive samples (Table 1), with 100% (432/432 bp) identity to the previously published sequence from this location (GenBank: KT725847). One sample contained DNA of *T. capreoli* isolate “capreoli CE-1”, with 100% (440/440 bp) identity to its sequence reported from Hungary (GenBank: KY308178).

### Piroplasms in stable flies from randomly chosen locations (localities 2–10)

Only 3.2% (5 of 157) of randomly collected stable flies were PCR-positive for piroplasms (Table 1). All these positive samples originated from places with horses nearby and contained DNA of *T. equi*, with 100% (303/303 to 437/437 bp) identities to previously reported sequences from Hungary (GenBank: JX4578282), Ukraine (GenBank: KP868757) and Switzerland (GenBank: KM046919, KM046920).

## Discussion

To our knowledge, this is the first account on the detection of *T. capreoli*, *T. equi* and *T. orientalis* DNA in *Stomoxys calcitrans*. Relevant to these results, biting flies were found negative for *T. orientalis* in Australia in a previous study [13], whereas mosquitoes were reported to carry this piroplasm in the UK [14]. Unexpectedly, no association was found between tick or stable fly infestation of horses and their *T. equi* seropositivity in Brazil [15].

Considering the preparation of stable flies for molecular analysis in this study, surface disinfection must have effectively removed the blood, which possibly contaminated the mouthparts outside. In addition, we hypothesize that the survival rate of a protozoan parasite (sensitive to desiccation) should be very low on such a surface. On the other hand, surface disinfection could not completely clear the inside/contents of tubular mouthparts, which remained always PCR-negative in this study. In line with this, it was postulated that the survival time of a protozoan parasite is probably very short even within the mouthparts of *S. calcitrans* [16]. Therefore, based on the present results (i.e. *T. orientalis* DNA absent in the head and mouthparts, but restricted to the thoracic-abdominal part of the flies), mouthpart-associated immediate transmission of theileriae by *Stomoxys* appears to be significantly less likely, than delayed transmission *via* regurgitated crop contents. This is in line with previous observations that the stable fly has a peculiar capacity for the delayed transmission of another protozoan parasite, *Trypanosoma evansi* [1].

The source of piroplasm DNA in the thorax-abdomen (as observed here) is supposed to be, at least partly, the diverticulum (crop) of stable flies, involved in regurgitation. In general, during the feeding of muscoid flies fluids that are dilute (e.g. blood) are shunted for temporary storage to the diverticulum; then, contents of the diverticulum from the previous blood meal are regurgitated through the proboscis into the feeding substrate [17]. Experimental evidence has shown that stable flies, in particular, will direct some of the ingested blood into their crop, where it can stay 24 hours or more [1]. Pathogens may survive longer in the crop than in the gut, because it is a more friendly environment devoid of digestive secretions [1]. Stable flies will then regurgitate part of the previous blood meal during the early phase of the next blood meal, before taking up a new one [18]. The volume of blood regurgitated by *S. calcitrans* is up to 0.18 μl [19], which (based on normal values of cattle) may contain more than 10^6^ bovine red blood cells. Thus, regurgitation of a relatively high amount of the previous blood meal could be an important way of transmitting high doses of disease agents [1].

Although the volume of ingested blood does not show significant difference between the sexes [20], females typically ingest blood meals an average of 1.8 times per day, and males 2.8 times per day [21]. This may explain why male stable flies of the present study carried piroplasm DNA significantly more frequently, implying that their suspected mechanical vector role (if any) may also surpass that of females.

There was no difference between the rate of PCR positivity among flies in the stable (which was void of cattle for at least two hours) and the pen on the pasture (where the animals were present at the time of sampling). This implies that PCR positivity did not depend on the presence of cattle next to the flies (i.e. on fresh blood meal). This was confirmed by finding one *T. capreoli*-positive sample, without roe deer on the pasture (and knowing that cattle are not susceptible to the roe-deer specific isolate “capreoli-CE1” detected here [22]). On the other hand, the low level of *T. equi* positivity in case of randomly collected samples suggests that the (former or nearby) presence of infected, piroplasm carrier animals is a prerequisite for any risks associated with the suspected vector role of stable flies.

Regarding the three *Theileria* species detected in *S. calcitrans*, *T. equi* is known to be transmitted mechanically *via* needles/syringes not exchanged between horses during blood sampling [23]. Members of the *T. orientalis* complex can also be transmitted mechanically through the inoculation of infected blood [5] or *via* horse flies [6] and biting lice [7]. It was suggested that further blood-sucking arthropod vectors of *T. orientalis* might exist, including biting flies [24], and stable flies in particular [25]. The chances of this is further enhanced by the generally long-term persistence of theileriae in the blood stream [26], similarly to another intra-erythrocytic bovine pathogen, *Anaplasma marginale*, of which ticks are biological vectors, but which may also have stable flies as mechanical vectors [27].

Results of the present study also reflected identity of all obtained *T. orientalis* sequences, and local genetic homogeneity of *T. orientalis* is thought to be interrelated with the absence of sexual recombination in ticks as biological vectors, i.e. the predominance of mechanical transmission [5]. *Haemaphysalis punctata* is the most likely tick vector of the *T. orientalis* complex in Europe and Hungary [12]. Larvae and nymphs of this tick species do not typically infest cattle [28, 29], thus their substantial role in the transstadial transmission, characteristic of theileriae, cannot be postulated. This raises the possibility and “ecological need” of *T. orientalis* for alternative modes of vector-borne transmission, most likely by blood-sucking flies.

## Conclusions

*Theileria* DNA is detectable in stable flies, in case of *T. orientalis* at least for two hours after blood-feeding, and in case of *T. capreoli* also in the absence of infected hosts (i.e. roe deer). Male flies rather than females, and thoracic-abdominal (most likely crop) contents rather than mouthparts may pose a risk of mechanical transmission. These data suggest that it is worth to study further the vector role of stable flies in the epidemiology of theilerioses, in which not the immediate, but rather the delayed transmission type seems possible.

## Data Availability

The sequences obtained and/or analyzed during the present study are deposited in the GenBank database under the accession numbers MN611176-MN611178. All other relevant data are included in the article; raw data are available upon request.
